# Deficiency in type 1 insulin-like growth factor receptor in mice protects against oxygen-induced lung injury

**DOI:** 10.1186/1465-9921-6-31

**Published:** 2005-04-08

**Authors:** Karmene Ahamed, Ralph Epaud, Martin Holzenberger, Monique Bonora, Jean-François Flejou, Julien Puard, Annick Clement, Alexandra Henrion-Caude

**Affiliations:** 1 INSERM U719, Hospital Saint-Antoine, 75012 Paris, France; 2 INSERM U515, Hospital Saint-Antoine, 75012 Paris, France; 3 Department of Pathology, Hospital Saint-Antoine, 75012 Paris, France

## Abstract

**Background:**

Cellular responses to aging and oxidative stress are regulated by type 1 insulin-like growth factor receptor (IGF-1R). Oxidant injury, which is implicated in the pathophysiology of a number of respiratory diseases, acutely upregulates IGF-1R expression in the lung. This led us to suspect that reduction of IGF-1R levels in lung tissue could prevent deleterious effects of oxygen exposure.

**Methods:**

Since IGF-1R null mutant mice die at birth from respiratory failure, we generated compound heterozygous mice harboring a hypomorphic (*Igf-1r*^*neo*^) and a knockout (*Igf-1r*^-^) receptor allele. These IGF-1R^neo/- ^mice, strongly deficient in IGF-1R, were subjected to hyperoxia and analyzed for survival time, ventilatory control, pulmonary histopathology, morphometry, lung edema and vascular permeability.

**Results:**

Strikingly, after 72 h of exposure to 90% O_2_, IGF-1R^neo/- ^mice had a significantly better survival rate during recovery than IGF-1R^+/+ ^mice (77% versus 53%, *P *< 0.05). The pulmonary injury was consistently, and significantly, milder in IGF-1R^neo/- ^mice which developed conspicuously less edema and vascular extravasation than controls. Also, hyperoxia-induced abnormal pattern of breathing which precipitated respiratory failure was elicited less frequently in the IGF-1R^neo/- ^mice.

**Conclusion:**

Together, these data demonstrate that a decrease in IGF-1R signaling in mice protects against oxidant-induced lung injury.

## Background

Critical-care patients, such as premature newborns or adults suffering from severe respiratory illness, often need high concentrations of oxygen to maintain adequate blood oxygenation. Yet, high oxygen concentrations for extended periods of time are toxic and can lead to impaired lung function, respiratory failure and even death [1-3]. The sequence of events resulting in acute lung injury has been described in different models and involves alveolar edema, surfactant dysfunction, interstitial fibrosis [4-6], and subsequent deterioration of lung function [7]. At the cellular level, hyperoxia generates oxygen-derived free radicals that lead to damaged lung parenchymal, epithelial and endothelial cells [3].

Insulin-like growth factor-I (IGF-I) and its cognate receptor, the type 1 insulin-like growth factor receptor (IGF-1R), are known to be key modulators of growth and injury repair processes in several organs, including the lung. IGF-1R is essential for normal growth, development and differentiation, and mediates signals for the suppression of apoptosis and promotion of mitogenesis [8]. Interestingly, we [9] and others [10] have observed that IGF-1R expression was more intense in areas of increased proliferation in the developing lung of rodents. Furthermore, exposure of adult rats to prolonged hyperoxia was associated with an increase in IGF-1R immunostaining in the lung, in the vicinity of cells expressing IGF-I [11]. Likewise, the expression pattern of IGF-I and IGF-1R in the lung is altered in various diseases that require oxygen therapy, including cystic fibrosis [12,13], lung fibrosis [14-16], acute respiratory distress syndrome or bronchopulmonary dysplasia [14]. Together, these observations have led to the suggestion that IGF signaling may be important in the pathogenesis of oxygen-induced lung injury.

Interestingly, a link between IGF signaling and resistance to oxidative stress was found in studies on the biological mechanisms of aging. Holzenberger *et al. *recently showed that mutant mice with only one functional copy of the *Igf-1r *gene had an extended lifespan [17]. Remarkably, increased longevity in these mutants was associated with enhanced resistance to paraquat-induced oxidative stress, while there was a slight reduction in body growth and no alteration in metabolism, food intake, body temperature or onset of sexual maturation and fertility. These findings suggest that the final common pathway of IGF-1R action in the aging process may be modulation of the cellular response to oxidative damage.

In this study, we sought to determine whether decreased functional levels of IGF-1R could prevent deleterious effects of oxygen exposure in the lung. Since IGF-1R null mutant mice die at birth from respiratory failure, we generated compound heterozygous mice harboring a hypomorphic (*Igf-1r*^*neo*^) allele and a knockout (*Igf-1r*^-^) allele [18]. These IGF-1R^neo/- ^mice, strongly deficient in IGF-1R, were subjected to hyperoxia and analyzed for survival time, ventilatory function, pulmonary histopathology, morphometry, lung edema and vascular permeability.

## Methods

### Animals

The mice were studied according to European recommendations on animal ethics. They were kept under standard conditions (average ambient temperature 23°C, 12/12-h light/dark cycle) with water and food provided *ad libitum *throughout the study. Experiments were performed on male and female mice with a 129/Sv genetic background. They were either deficient in IGF-1R (IGF-1R^neo/-^) or had normal levels of IGF-1R (IGF-1R^+/+^). The hypomorphic IGF-1R^neo ^allele was created by introducing a neomycin resistance cassette (neo) in intron 2 of the receptor gene using homologous recombination, and the receptor null allele (IGF-1R^-^) resulted from the complete elimination of exon 3, which encodes the type 1 IGF receptor ligand-binding domain [18]. We compared heterozygous IGF-1R^neo/- ^mice (n = 78) with their wild-type IGF-1R^+/+ ^littermates (n = 84), which served as controls. Body weight and body temperature were systematically measured prior to each experiment. Seven-week-old mice were analyzed at three different time periods: under basal conditions, after hyperoxic exposure and/or during post-hyperoxia recovery. Genotyping of the mice was performed by PCR. Amplification conditions were as follows: 95°C for 3 min, and 35 cycles at 95°C for 35 s, 55°C for 45 s and 72°C for 1 min, with a final elongation step at 72°C for 7 min. To detect *IGF-1R*^*neo *^(hypomorphic), *IGF-1R*^- ^(knockout) and *IGF-1R*^+ ^(wild-type) alleles simultaneously, three oligonucleotides were used. The primer sequences were 5'-CATGGGTGTTAAATGTTAATGGC-3' (Nex, sense-oriented), 5-ATGAATGCTGGTGAGGGTTGTCTT-3' (Nvl, sense-oriented) and 5'-ATCTTGGAGTGGTGGGTCTGTTTC-3' (Nmt2, antisense-oriented).

### Hyperoxic exposure and post-hyperoxia recovery

To induce hyperoxic lung injury, mice were exposed to 90% O_2 _for 72 h in a sealed Plexiglas chamber of 9.7 liters, at 22–24°C. The O_2 _and CO_2 _levels in the chamber were monitored by O_2 _and CO_2 _analyzers (OM-11 and LB-2, respectively; Beckman Instruments, Schiller Park, IL, USA). Oxygen flow rate was adjusted to 1.5 L/min. The CO_2 _level was always kept below 0.5%. Following hyperoxia, the mice were then placed in normoxic basal conditions. The survival study was performed in 31 IGF-1R^neo/- ^and 30 IGF-1R^+/+ ^mice. They were checked every 2 h.

### Ventilatory physiology

Analyses were performed sequentially in the same 5 IGF-1R^neo/- ^and 6 IGF-1R^+/+ ^mice. There were three experimental conditions: before hyperoxia (in the basal, normoxic conditions), after hyperoxia, and during the post-hyperoxia recovery. Ventilatory parameters were recorded in awake and unrestrained mice placed in a whole-body plethysmograph, by using the barometric method [19]. The pressure signal resulting from breathing was detected using a differential pressure transducer (Validine DP103/12; Validine, Northridge, CA, USA) connected to the animal chamber (400 mL) and to a reference chamber of the same volume. The spirogram was recorded and stored on a computer using respiratory acquisition software (CIO-DAS 1602/16 interface and ELPHY software) for analysis off-line. Calibration was performed at the beginning of experiments by several injections of 50 μL air into the chamber. Each animal was weighed and placed in the chamber. A thermistal probe (BIO-BIT14) was inserted rectally and secured in place at the base of the tail and on the wall of the chamber. A protective muff was placed around the mouse to prevent stress. The protocol consisted of recording ventilatory parameters and rectal temperature before and immediately after the 72 h of hyperoxia. Measurements were made every 5 min while the mouse was breathing the following gas mixtures: 20 min in hyperoxia (100% O_2_) to obtain baseline values, 20 min in normoxia (21% O_2_), 10 min in mild hypoxia (12% O_2_) and 10 min in severe hypoxia (10% O_2_). The mouse was then allowed to recover in normoxia for 24 h prior to changing the experimental conditions, and the same protocol of recording was again applied. The CO_2 _concentration in the chamber was always < 1% at the end of each session. The following variables were measured and calculated by a computer-assisted method: minute ventilation (V_E_) and its two components: tidal volume (V_T_) and respiratory frequency (f_R_). For each 5 min recording, values were averaged on 50–100 contiguous breaths.

### Measurement of relative lung weight and lung histology analysis

Mice were weighed and anesthetized with sodium pentobarbital (60 mg/kg). After bleeding, the lung was either immediately removed to measure the relative lung weight, or perfused *in situ *with neutral-buffered 10% formalin (15 cm H_2_O pressure) for histological analysis. The ratio of lung weight to body weight was determined in 11 IGF-1R^neo/-^and 16 IGF-1R^+/+ ^mice under basal conditions.

For histological analysis, the trachea was ligated, the lungs were removed and fixed overnight in 10% formalin, and embedded in paraffin. Lung histology was documented both under basal conditions and after hyperoxic exposure, using 4 IGF-1R^neo/- ^and 4 IGF-1R^+/+ ^mice for each condition. Three random 4-μm paraffin-embedded tissue sections from four different lungs from each group were stained with hematoxylin-eosin. The histopathology was reviewed in a blinded manner with respect to which group or mouse was being reviewed, using characteristic pathological traits including: alveolar destruction, edema, hemorrhage and hyaline membrane deposition.

Morphometric analysis was performed under basal conditions and after hyperoxic exposure, using 4 IGF-1R^neo/- ^and 4 IGF-1R^+/+ ^mice for each condition. Images were captured using the software TRIBVN ICS (release 2.04; IMAGIC, Bildverarbeitung AG, Glattbrug, Zürich, Switzerland). Six randomly selected fields (magnification 20×) were assessed in each mouse for (i) intra-alveolar hemorrhage and (ii) fibrin deposition which are easily identifiable pathological processes. Each process was scored on a scale of 0 to 4. Scores were assessed twice in a blinded manner. Zero with no apparent injury; 1, mild injury with <25% lung involvement; 2, moderate injury with 25–50% lung involvement; 3, severe injury with 50–75% lung involvement; 4, very severe injury with >75% lung involvement. An overall score of hyperoxia-induced lung injury was obtained by summation of scores of hemorrhage and of fibrin deposition. A mean ± SEM was generated from each group. In addition, five fields from each mouse were selected for the presence of perivascular edema, and used to quantify the edema by the ratio between the surface of edema and the surface of the vessel. Surface was estimated using a one-dimensional grid of points spaced 50 μm apart which was overlaid on each field.

### Lung edema analysis and vascular permeability by Evans Blue assay

Pulmonary edema was determined from the ratio between fresh lung weight at autopsy and dry weight. Lungs were dried at 80°C until their weight remained constant over 24 h. The wet-to-dry lung weight was assessed under basal conditions using 4 IGF-1R^neo/- ^and 4 IGF-1R^+/+ ^mice, and after hyperoxic exposure using 8 IGF-1R^neo/- ^and 7 IGF-1R^+/+ ^mice for each experiment.

Vascular permeability related to lung injury was measured using the Evans Blue dye extravasation assay, as previously described [20]. The animals were anesthetized with a combination of ketamine (24 mg/kg) and xylazine (36 mg/kg), and received 30 mg/kg Evans Blue dye dissolved in 0.9% saline at a final concentration of 20 mg/ml by jugular vein injection, 10 min before sacrifice. The lungs were then perfused with 5 ml PBS containing 5 mM EDTA until all the blood has been removed. They were then dissected out, weighed and incubated at room temperature for 24 h in 4 ml formamide per g tissue (Sigma, St. Louis, MO, USA). The extravasation of Evans Blue-labeled albumin from the tracheobronchial microcirculation was quantified by measuring the optical density (OD) of the formamide extracts at wavelength of 620 nm. The quantity of Evans Blue dye extravasated in the airway tissues, expressed in ng/mg of dry tissue weight, was interpolated from a standard curve of Evans Blue concentrations (0.5–10 μg/ml). The Evans Blue dye assay was assessed both under basal conditions using 5 IGF-1R^neo/- ^and 6 IGF-1R^+/+ ^mice, and after hyperoxic exposure using 6 IGF-1R^neo/- ^and 7 IGF-1R^+/+ ^mice for each experiment.

### Statistical analysis

The values for all animals within each experimental group were averaged and standard deviations (SD) calculated. Statistical comparison of the results was performed between IGF-1R^+/+ ^and IGF-1R^neo/- ^mice, and between normoxia and hyperoxia-treated groups, using the unpaired ANOVA *t*-test and the multiple comparison test (Bonferroni). The significance between the survival rate of two groups was determined by Kaplan-Meier analysis using the Cox statistical test. Differences were considered significant if *P *< 0.05.

## Results

### Survival of compound IGF-1R^neo/- ^mice

**Figure 1:**
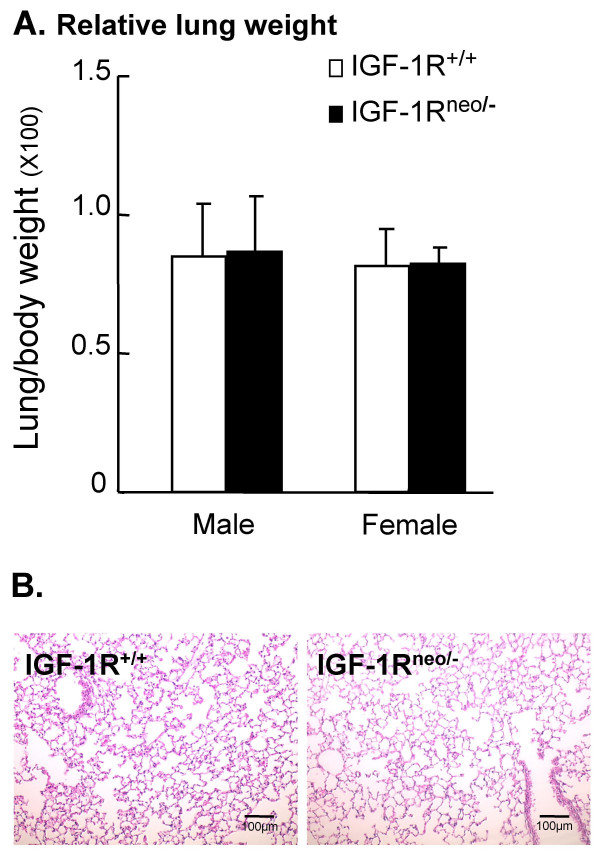
**Lung weight and histology under basal conditions. **(A) Mean values (×100) of lung-to-body weight ratio (± SD) in IGF-1R^+/+ ^mice (male: n = 11; female: n = 5) and IGF-1R^neo/- ^mice (male: n = 7; female: n = 4). (B) Representative histology of hematoxylin-eosin stained lung sections from IGF-1R^+/+ ^and IGF-1R^neo/- ^mice. Magnification: 10× objective.

To assess the importance of IGF-1R for postnatal lung function, compound mice that harbored a hypomorphic (*Igf-1r*^*neo*^) allele and a knockout (*Igf-1r*^-^) allele were generated. As previously described [21], IGF-1R^neo/- ^mice constitutively express only 25% of the IGF-1R levels typically found in wild-type (IGF-1R^+/+^) mice. In the IGF-1R^neo/- ^mice, survival up to 6 months and fertility in young adults were unaltered (data not shown). Body weight of IGF-1R^neo/- ^and IGF-1R^+/+ ^female mice did not differ significantly (-3.8%, NS), while the body weight of IGF-1R^neo/- ^males was slightly reduced compared to IGF-1R^+/+ ^littermates (-16.6%, *P *< 0.05). In both female and male mice, the lung to body weight ratio was similar in both genotypes (Fig. 1A). Under basal normoxic conditions, histological examination of the lungs of IGF-1R^neo/- ^and IGF-1R^+/+ ^mice did not reveal fibrosis, nor inflammation, nor remodeling of intrapulmonary airways and lung parenchyma (Fig. 1B).

**Figure 2:**
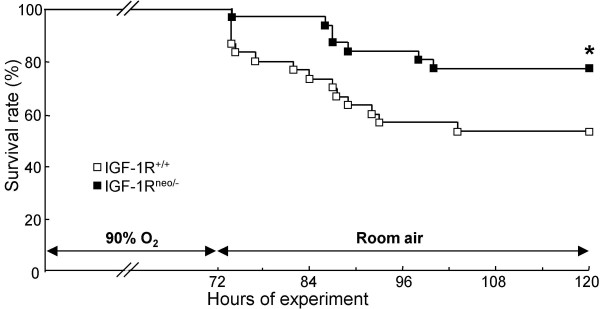
**Survival rate following exposure to 90% oxygen. **The survival times of IGF-1R^+/+ ^mice (male: n = 15; female: n = 15) and IGF-1R^neo/- ^mice (male: n = 16; female: n = 15) were measured following exposure to 90% oxygen. Data are expressed as percentage of mice alive at each time point. The asterisk indicates significant differences between IGF-1R^+/+ ^and IGF-1R^neo/- ^mice, as determined by Kaplan-Meier analysis; *P *< 0.05.

To investigate whether IGF-1R contributed to hyperoxia-induced lung injury, IGF-1R^neo/- ^mice and their wild-type littermate controls were exposed to 90% O_2 _for 72 h and their survival was assessed during a recovery period of 48 h under normoxic conditions (Fig. 2). Forty-eight hours actually corresponded to the critical period during which mice either developed lethal respiratory symptoms or survived. The percentage of survivors following the initial period of hyperoxia was significantly greater in the IGF-1R^neo/- ^mice (77%) than in IGF-1R^+/+ ^mice (53%) (*P *< 0.05). Moreover, mortality predominantly affected mice within the initial 24-h period after hyperoxia (Fig. 2).

### Ventilatory parameters in IGF-1R^neo/- ^mice

We recorded ventilation in conscious IGF-1R^neo/- ^and IGF-1R^+/+ ^mice placed in a whole-body plethysmograph while breathing 90% O_2_, 21% O_2 _or under hypoxia (at 12% and 10% O_2_). Baseline ventilation and control of breathing during hypoxia were assessed by measuring the respiratory parameters such as minute ventilation (V_E_) and its two components: respiratory frequency (f_R_) and tidal volume (V_T_) before hyperoxia, after hyperoxia and during recovery in room air.

**Figure 4:**
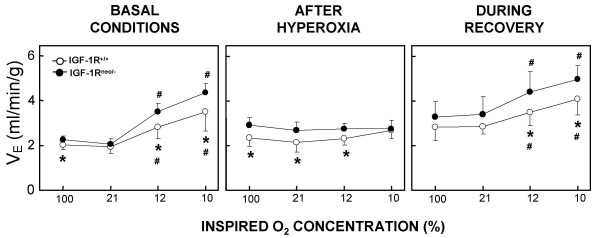
**Ventilatory responses before and after hyperoxia, and during recovery. **Minute ventilation (V_E_) in response to decreasing levels of inspired O_2_. In each one of the three conditions, mean values (± SD) of VE in IGF-1R^+/+ ^and IGF-1R^neo/- ^mice were recorded under 90% O_2_, 21% O_2 _and at two levels of hypoxia (12% and 10% O_2_). Values with asterisks are significantly different from control values at 90% O_2_. Crosses indicate significant differences from normoxic control values, and asterisks indicate significant differences between IGF-1R^+/+ ^and IGF-1R^neo/- ^mice; *P *< 0.05.

Before hyperoxia (control conditions), baseline minute ventilation (V_E_) was slightly greater in IGF-1R^neo/- ^mice than in IGF-1R^+/+ ^mice because of a greater tidal volume (V_T_), while respiratory frequency (f_R_) was similar in the two groups (Table 1). During hypoxia, V_E _significantly increased under 12% and 10% O_2 _in both groups, this effect being essentially due to a large increase in f_R _(Fig. 4). During this protocol, mean values of V_E _were constantly greater in IGF-1R^neo/- ^mice than in IGF-1R^+/+ ^mice. However, the magnitude of the response to hypoxia – which was obtained by calculating the per cent change in V_E _during severe hypoxia (10% O_2_) relative to hyperoxia – was similar in the two groups.

**Table 1:** Ventilatory parameters at baseline (90% O_2_) before hyperoxia, after hyperoxia and during recovery.

	IGF-1R^+/+^			IGF-1R^neo/-^		
		
	Before hyperoxia	After hyperoxia	During recovery	Before hyperoxia	After hyperoxia	During recovery
V_E_	2.03 ± 0.17	2.35 ± 0.32 ^#^	2.84 ± 0.69^#^	2.27 ± 0.22 *	2.93 ± 0.37 *^#^	3.30 ± 0.62^#^
V_T_	10.0 ± 0.5	15.8 ± 2.0^#^	14.4 ± 2.2^#^	11.2 ± 0.8 *	15.2 ± 1.1^#^	14.8 ± 1.1^#^
f_R_	203 ± 9	142 ± 30^#^	196 ± 32	203 ± 21	192 ± 18 *^#^	222 ± 35

Values are mean parameters ± SD in IGF-1R^+/+ ^mice (n = 6) and IGF-1R^neo/- ^mice (n = 5) mice; V_E_, minute ventilation expressed in ml/min/g, V_T_, tidal volume in μl/g, f_R_, respiratory frequency in c/min; # significant difference relative to control before hyperoxia; * significant difference between the two genotypes (*P *< 0.05).

**Figure 3:**
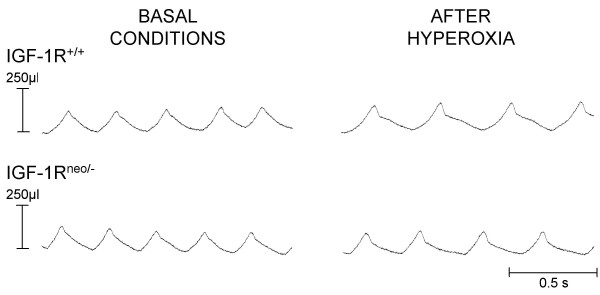
**Respiratory pattern before and after hyperoxia. **Typical respiratory pattern in a control IGF-1R^+/+ ^mouse (upper traces) and in an IGF-1R^neo/- ^mouse (lower traces) under basal conditions and following hyperoxia. Expiratory pauses were observed in most control mice but not in IGF-1R^neo/- ^mice.

Following hyperoxia, the mice of both genotypes manifested an abnormal pattern of breathing. This pattern was characterized by a deep and slow breathing with expiratory pauses, particularly in the IGF-1R^+/+ ^mice (Fig. 3). Mean values of baseline V_E _were substantially increased compared with control conditions in both groups, this effect being due to a large increase in V_T_, whereas f_R _was moderately decreased (Table 1). However, the hyperoxia-induced increase in V_E _was greater in IGF-1R^neo/- ^mice because of a less pronounced diminution of f_R_. During hypoxia, V_E _did not increase under 12% or 10% O_2 _in either of the genotypes, which indicates that prolonged hyperoxic exposure abolished the ventilatory response to hypoxia (Fig. 4).

During recovery in room air, V_E _and V_T _remained elevated while f_R _recovered its control values in both IGF-1R^neo/- ^and IGF-1R^+/+ ^mice (Table 1), and the stimulation of ventilation by hypoxia was restored (Fig. 4). Again, mean values of V_E _at 12% and 10% O_2 _were significantly greater in IGF-1R^neo/- ^mice than in IGF-1R^+/+ ^mice, but the magnitude of the response was similar in both groups.

### IGF-1R deficiency confers protection in 90% O_2_

**Figure 5:**
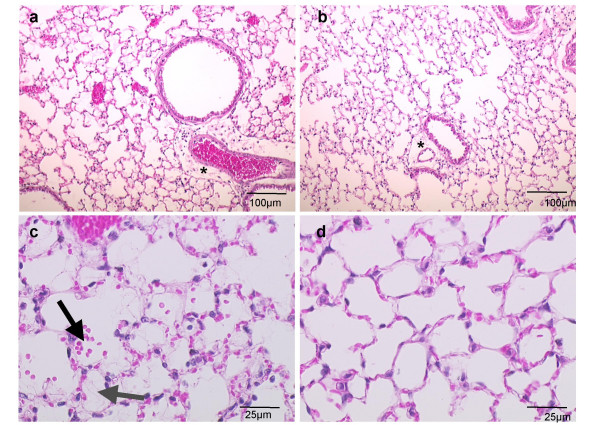
**Histology following hyperoxic injury. **Hematoxylin-eosin stained lung sections of (a) IGF-1R^+/+^, and (b) IGF-1R^neo/- ^mice following 72 h of hyperoxic exposure, illustrating perivascular and peribronchiolar edema as indicated by asterisks. Magnification: 10× objective. Representative histology of lung sections demonstrates (c) focal alveolar hemorrhages (black arrow) and hyaline membrane formation (gray arrow) in IGF-1R^+/+ ^lungs, and (d) minimal lesions in IGF-1R^neo/- ^mice. Magnification: 40× objective.

As shown in Fig. 5, exposure to 90% O_2 _for 72 h caused histological changes in the lungs of both IGF-1R^+/+ ^and IGF-1R^neo/- ^mice, but injury was more severe in the lungs taken from IGF-1R^+/+ ^mice. The histological lesions included alveolar destruction, hyaline membrane formation, hemorrhage, and perivascular and peribronchiolar edema (Fig. 5). In contrast, hyperoxia-induced lung lesions were remarkably milder in IGF-1R^neo/- ^mice, with less edema and hemorrhage.

**Figure 6:**
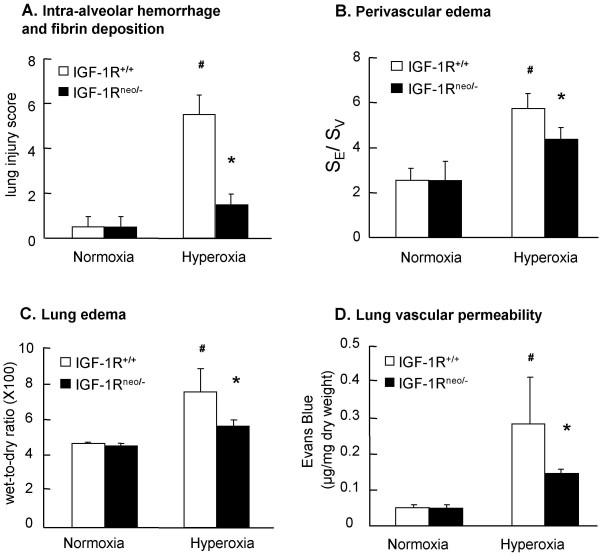
**Hyperoxia-induced lung injury, lung edema and vascular permeability. **Parameters of lung damage were considered under basal conditions and following hyperoxia. (A) Lung injury scores from IGF-1R^+/+ ^(n = 4) and IGF-1R^neo/- ^(n = 4) mice under basal conditions (normoxia) and under hyperoxia. A cumulative score was based on evaluation of hemorrhage and intraalveolar deposition (6 random sections per lung). (B) Quantitated perivascular edema from IGF-1R^+/+ ^(n = 4) and IGF-1R^neo/- ^(n = 4) mice under basal conditions (normoxia) and under hyperoxia. Surface of perivascular edema was reported to the surface of the vessel, and noted S_E_/S_V _(5 selected sections per lung). (C) Lung edema was determined as mean values (± SD) of wet-to-dry lung weight ratio under basal conditions (normoxia) in IGF-1R^+/+ ^(n = 4) and IGF-1R^neo/- ^(n = 4) mice, and under hyperoxia in IGF-1R^+/+ ^(n = 7) and IGF-1R^neo/- ^(n = 8) mice. (D) Evans Blue dye extravasation was assessed as mean values (± SD) in IGF-1R^+/+ ^(n = 13) and IGF-1R^neo/- ^(n = 11) mice. Crosses indicate significant differences between hyperoxic and normoxic values in IGF-1R^+/+ ^mice, and asterisks indicate significant differences between IGF-1R^+/+ ^and IGF-1R^neo/- ^mice under hyperoxic conditions; *P *< 0.05.

Lung morphometry was used to measure the degree of hyperoxic injury. Analysis of a cumulative score which reflects both intra-alveolar hemorrhage and fibrin deposition revealed a significantly reduced score in hyperoxic-exposed IGF-1R^neo/- ^mice as compared to IGF-1R^+/+ ^(Fig. 6A). Likewise, quantitative assessment of perivascular edema, as shown in Fig. 6B, confirmed that IGF-1R^neo/- ^were protected from hyperoxia induced lung injury, and indistinguishable from IGF-1R^+/+ ^mice under basal conditions.

We quantified lung damage in IGF-1R^neo/- ^and IGF-1R^+/+ ^mice using two parameters which are either relevant to edema or to vascular permeability (Fig. 6). Wet-to-dry lung weight ratio, which was measured as a supplementary mean to quantify edema, was noticeably reduced in IGF-1R^neo/- ^mice (-26%, *P *< 0.05) (Fig. 6C). This data was consistent with histomorphometric results (Fig. 6B). Moreover, lung vascular permeability, which was quantified by measuring the extravasation of Evans Blue dye, was reduced in IGF-1R^neo/- ^mice (-49%, *P *< 0.05) (Fig. 6D).

## Discussion

The main aim of the present study was to evaluate the importance of IGF-1R in acute hyperoxic lung injury by studying survival time, respiratory physiology and pulmonary histology. Since complete inactivation of *Igf-1r *is lethal at birth due to respiratory failure [18,22], we combined a knockout allele (*Igf-1r*^-^) and a hypomorphic allele (*Igf-1r*^*neo*^) in order to generate viable mice with markedly reduced levels of IGF-1R [21]. Although haploinsufficiency of the *Igf-1R *gene in humans has been suspected to be associated with lung hypoplasia [23], the low levels of IGF-1R expressed in IGF-1R^neo/- ^mice were sufficient to ensure normal lung growth (Fig. 1). Thus, these mice provided a new way of exploring the *in vivo *effects of a major deficiency in IGF-1R in the adult lung. The most striking result of this study was the improved survival of the IGF-1R^neo/- ^mutant mice following hyperoxic injury, associated with increased protection against pulmonary damage. These results support the idea that IGF-1R plays a critical role in the regulation of pulmonary resistance to oxidative stress.

Recently, using paraquat-induced stress which is known to cause multi-organ failure, Holzenberger *et al. *have shown that deficiency in IGF-1R is associated with improved oxidative stress resistance, as assessed by prolonged survival [17]. In this study, we further evaluated IGF-1R as one component of the mechanistic determinants of the lung injury process, and focused our attention on survival after hyperoxic-lung injury. IGF-1R^neo/- ^mutant mice and their wild-type littermate controls were exposed to hyperoxia-induced oxidative stress, which has been used extensively in rodents as a valuable model of acute respiratory distress. Because sensitivity to hyperoxia has been described to be strain-dependent [24], these experiments were performed using IGF-1R^- ^and IGF-1R^neo ^alleles in a pure 129/Sv genetic background. Consistent with previous findings [25], acute respiratory distress was observed in our mice when they were exposed to > 90% oxygen for 72 h. Overall, IGF-1R^neo/- ^mice were more resistant to hyperoxic stress than littermate controls (Fig. 2,3,4,5,6). These results, together with the induction of IGF-1R in response to high oxygen concentration [11,14], directly implicate IGF-1R in the development of oxidant-mediated lung injury, and indicate that deficiency in IGF-1R is associated with a higher capacity to endure and to recover from oxidant-induced injury. There are numerous mechanisms that can potentially interfere with resistance to hyperoxia, involving antioxidant enzymes, inflammatory mediators, cell cycle progression regulators, apoptosis and anti-apoptosis factors, endothelial cell-specific factors and the extracellular matrix repair system. Although assessment of the importance of IGF-1R in each of these mechanisms warrants specific attention, we made it our priority to determine which of the physiological traits in these mice is most likely to account for the phenotypic differences in response to acute lung injury.

Hyperoxic exposure is known to induce severe lung dysfunction, which can dramatically affect gas exchange. Indeed, several studies have shown that prolonged hyperoxia causes a reduction in lung compliance [26,27], and markedly alters the control of breathing through attenuating both carotid chemosensory responses and chemoreflex responses to hypoxia [28]. We hypothesized that such alterations may influence the resistance to oxidative stress, and subsequent survival of the mice. We found that hyperoxic exposure of the mice of either genotype, IGF-1R^neo/- ^and IGF-1R^+/+^, abolished the ventilatory response to hypoxia, mainly caused by the absence of responsiveness of respiratory frequency (Fig. 4). These results clearly suggest that prolonged hyperoxia markedly alter carotid chemosensory activity and are consistent with previous studies in unanesthetized cats [1] and rats [29]. Interestingly, we observed also that the deleterious effects of hyperoxia on the hypoxic ventilatory drive were fully reversible under recovery conditions in room air. Thus, the blunted ventilatory response to hypoxia immediately after hyperoxic injury probably plays a critical role in the development and progression of respiratory distress exhibited by the mice.

It is noteworthy that striking differences in pattern of breathing and ventilation were observed between IGF-1R^neo/- ^and IGF-1R^+/+ ^mice, which may well contribute to the improved survival of the former group after hyperoxia. In particular, the abnormal pattern of breathing induced by hyperoxia, which limited the ventilatory response to hypoxia and precipitated respiratory failure, was more often elicited in the control mice than in the IGF-1R^neo/- ^mice (Fig. 3). Also, minute ventilation at baseline, as well as in response to hypoxia, was greater in the IGF-1R^neo/- ^mice. This latter finding suggests a pulmonary role for IGF-1R in response to hypoxia. This is consistent with a previous observation in which IGF-1R mRNA levels were specifically increased by 200% in the lung in response to hypoxia [30].

Pathogenesis of lung injury after hyperoxia has been studied extensively in mice. Prominent features include extensive alveolar and endothelial cell death, leading to disruption of the alveolo-capillary barrier, high pulmonary microvascular permeability and pulmonary edema. The hyperoxic morphological phenotype that we quantified by morphometry at 72 h of hyperoxia was consistently characterized by pulmonary edema, intra-alveolar hemorrhage and hyaline membrane formation in IGF-1R^+/+^, but was remarkably milder in the IGF-1R^neo/- ^mice (Figs. 5 and 6). These results are directly supportive of decreased sensitivity to hyperoxia being associated with IGF-1R deficiency. Protective mechanisms in which IGF-1R is suspected to interfere may include changes in the defense against accumulation of oxygen free radicals, although a recent study in patients with respiratory distress syndrome or bronchopulmonary dysplasia indicated only moderate induction of the antioxidant defense system [31]. Likewise, the response of mice to prolonged hyperoxia was not associated with altered expression of classical antioxidant enzymes such as catalase, MnSOD and Cu-Zn SOD [32]. Other differences may reside in the inflammatory response that leads to an influx of protein-rich fluid to the airspaces.

By measuring changes in the ratio of wet-to-dry lung weight and in the Evans Blue extravasation, we found decreased permeability of the alveolo-capillary membrane in the lungs of IGF-1R^neo/- ^mice compared with IGF-1R^+/+ ^mice (Fig. 6). These results further led to consider that IGF-1R may exert its influence on the integrity of alveolo-capillary membrane through endothelial cells. Increased integrity of the alveolo-capillary membrane in the mutant mice may result from enhanced survival and/or proliferation of the alveolar and endothelial cells in response to oxidative stress [10]. Studies have indicated that IGF-1R is present in vascular endothelium and vascular smooth muscle of oxygen-exposed lungs, both in patients and in animal models [11,14]. To date, most studies on the regulation of vascular permeability have focused on vascular endothelial growth factor (VEGF) and related molecules. In the lung, however, their role in the control of permeability remains controversial [33-35]. Moreover, *in vivo *studies on the retinal neovascularization process have shown that VEGF is regulated through the activation of IGF-1R. This was demonstrated using either IGF-1R neutralizing antibody [36] or an endothelial cell-specific knockout of IGF-1 receptor [37]. Thus, it can be speculated that the deficiency of IGF-1R in IGF-1R^neo/- ^mice contributes to the protection of lung endothelium from oxidant injury – either directly or through intermediary modulation of VEGF expression. Taken together, these findings highlight the need for additional experiments to elucidate the true role of IGF-1R in the regulation of pulmonary permeability.

## Conclusion

Understanding of the molecular mechanisms allowing protection against pulmonary oxygen toxicity is crucial in order to enable the development of targeted and effective interventions aimed at preventing or alleviating oxidant-induced acute lung injury [38]. Our results support the novel notion that IGF signaling, for which numerous interfering drugs are under clinical development [39], plays an important role in the sensitivity to and recovery from oxygen lung injury. We consider that the findings of our study will precipitate additional research efforts to assess whether interference with IGF-1R signaling may be a way of reducing or preventing the development of acute respiratory distress syndrome.

## Competing interests

The author(s) declare that they have no competing interests.

## Authors' contributions

KA conceived of the experiment and carried out all animal studies. RE conceived of the design of the study, assisted in the acquisition of data and helped to draft the manuscript. MH provided the mouse model of IGF-1R deficiency, provided expert advice, and critically revised the manuscript. MB performed all ventilatory measurements and helped to draft the manuscript. JFF performed the histological analyses and provided expert advice and interpretation of the results. JP carried out the design of hyperoxic exposure. AC participated in the direction of the study and critically revised the manuscript. AHC conceived of the study and participated in its design and coordination, drafted and edited the manuscript. All authors read and approved the final manuscript.
